# Chemical crystal identification with deep learning machine vision

**DOI:** 10.1186/s13104-018-3813-8

**Published:** 2018-10-05

**Authors:** Perseverança Mungofa, Arnold Schumann, Laura Waldo

**Affiliations:** Soil and Water Science Department, Citrus Research and Education Center, University of Florida, 700 Experiment Station Rd, Lake Alfred, FL 33850 USA

**Keywords:** Deep learning, Image classification, Chemical crystals, GoogLeNet, VGG-16, Microscopic objects

## Abstract

**Objective:**

This study was carried out with the purpose of testing the ability of deep learning machine vision to identify microscopic objects and geometries found in chemical crystal structures.

**Results:**

A database of 6994 images taken with a light microscope showing microscopic crystal details of selected chemical compounds along with 180 images of an unknown chemical was created to train and test, respectively the deep learning models. The models used were GoogLeNet (22 layers deep network) and VGG-16 (16 layers deep network), based on the Caffe framework (University of California, Berkeley, CA) of the DIGITS platform (NVIDIA Corporation, Santa Clara, CA). The two models were successfully trained with the images, having validation accuracy values of 97.38% and 99.65% respectively. Finally, both models were able to correctly identify the unknown chemical sample with a high probability score of 93.34% (GoogLeNet) and 99.41% (VGG-16). The positive results found in this study can be further applied to other unknown sample identification tasks using light microscopy coupled with deep learning machine vision.

## Introduction

The recent advances in computer technology have allowed an evolution of machine vision with deep learning artificial neural networks, making it possible to obtain accurate results of image analysis and object recognition in near real time. Deep learning machines are very powerful systems for data analysis that integrate a set of techniques used to identify objects through images, translate speech into text, expose products of interest to a group of users, and compile relevant information [1]. Recently, the deep convolutional neural networks (CNN) have shown exceptional performance in the image classification and object detection tasks, due to their deeper and wider multilayers grouped in convolutions that improves accuracy while making efficient use of computer resources and reducing processing time [1, 2].

This research was conducted to test the ability of deep learning machine vision to identify chemical crystals by their characteristic microscopic structure and geometries, with the prospect of later using this method to identify microscopic pests and pathogens of food crops. Ziletti et al. [3] reported machine learning techniques for identifying chemicals from their crystal lattice structure patterns, which in turn were obtained from X-ray diffraction. Our study involves identification of crystal structure from light microscopy, not X-ray diffraction, and we could not locate previous studies where this light microscopy approach had been successfully implemented. A similar approach was previously implemented by Fuentes et al. [4] to develop a pest and disease detection system, using a deep learning-based detector.

In this study, eleven known chemical crystal samples were used to train two deep learning models for image classification: GoogLeNet and VGG-16, winning the first and second position at the ImageNet Large-Scale Visual Recognition Challenge in 2014 (ILSVRC14), respectively [5]. The trained models were used to identify an unknown chemical found in an unmarked container in one of our agricultural research facility’s laboratories. The methodology developed in this research may be later replicated in other research cases for the identification of microscopic objects and organisms.

## Main text

### Methods

The research was carried out at the Soil and Precision Agriculture Laboratory at the University of Florida, Citrus Research and Education Center, (https://crec.ifas.ufl.edu/). A deep learning machine vision approach for image classification was applied to train two deep CNNs loaded with the weights of pre-trained models (GoogLeNet and VGG-16). The method of using pre-trained model weights rather than zero starting weights, greatly improves the rate of network learning during training. In this case, the networks were trained to recognize geometric structures of chemical crystals and then identify an unknown chemical, through images captured by a digital microscope.

#### Experiment and data collection

The unknown chemical was a colorless prilled, water-soluble, odorless material, which we suspected to be a fertilizer. Based on that assumption, eleven chemical compounds (common agricultural fertilizers and chelates) were carefully selected to create the crystal images database for training neural networks. Sucrose was included as a second organic compound to compare with fertilizer urea, and potassium bromide was added to contrast with closely related potassium chloride fertilizer.

A 1 M solution of each chemical was prepared using distilled water, then six drops of solution were added on a microscope slide, separated into three sections of two drops each. A total of 210 slides were prepared per chemical, and three photos were taken per slide (one photo per section), for total number of approximately 630 photos per chemical. Before photography, the samples were subjected to evaporation and crystallization in a drying oven at 30 °C, for a minimum period of 3 h, except for citric acid and sucrose, whose drying temperature were 55 °C for 7 h and 50 °C for 5 h, respectively. Special attention was given to hygroscopic chemicals: ammonium nitrate, urea and potassium nitrate, which were maintained in the oven (at 30 °C), to avoid the absorption of humidity and to preserve characteristics of the crystals.

The images were taken using a color digital microscope camera of 9 megapixels. All images were in RGB format, with a resolution of 3488 × 2616 pixels and region of interest (ROI) of 2300 × 1400 pixels covering the major area containing valid information in the image. The microscope magnification was set to 2.25× with a field of view of 8.9 mm. Light balance (contrast and brightness) was adjusted when necessary to improve features visibility.

#### Methodology for sample preparation of the unknown chemical

In order to prepare samples of the unknown chemical at similar ranges of concentrations to the 11 known chemicals, three solutions of the unknown chemical were prepared, based on the averaged molecular weight of the 11 known chemicals and respective standard deviation. The averaged molecular weight was 145.648 g/mol and the calculated standard deviation value was 84.998. The dummy molar weight calculated for each repetition of the unknown sample is indicated below:

- Repetition 1 (average) = 145.648 g/mol.
- Repetition 2 (average − Std.) = 60.65 g/mol.
- Repetition 3 (average + Std.) = 230.646 g/mol.

The solutions were prepared following the procedure previously described and subjected to evaporation and crystallization for 3 h at 30 °C. Each repetition had 60 images and a total number of 180 images were used to test the model’s performance in image classification.

#### Data analysis

Image classification and probability analysis for object recognition were performed on a Linux server equipped with a graphics processing unit (GPU). We used two CNN models: GoogLeNet, a 22 weight layers, deep and wide CNN model with improved computation efficiency introduced by Szegedy et al. at the ILSVRC14 [6], and VGG-16, a 16 deep weight layers model, mostly used as feature extractor for image classification and object identification [7]. The two models are based on the Caffe framework of the DIGITS platform (Deep Learning GPU Training System) of the NVIDIA GPU [8]. Image classification probability results from GoogLeNet and VGG-16 models were analyzed by analysis of variance and Duncan’s multiple range test using GenStat (VSN International, Hemel Hempstead, UK).

#### Training methodology for GoogLeNet and VGG-16

The training process allows the system to recognize, differentiate and classify the dataset being analyzed and finally identify any unknown data based on supplied information. In this work, both models were trained with the AdaDelta algorithm, a gradient-based optimization method [9]. The base learning rate was set to 0.1 using an exponential decay function [10]. By default, the total number of training epochs was 30 with one snapshot per interval and one validation interval.

All images in the database were used for training and validation, subdivided into two sets of 75% for training and 25% for validation. The images were first resized by the DIGITS software to a fixed resolution of 256 × 256 pixels. Two data augmentation methods were applied to the training set by the DIGITS software. The first was image translation and horizontal reflections or flipping, and the images were cropped by the DIGITS software to the size of 224 × 224 pixels (the image input size used inside the convolutions) and a mirror-image copy was generated. The second was RGB color shift to alter RGB intensity in the images [6, 7, 11].

For the unknown test sample analysis, three repetitions of 60 unknown images were used and the input image size for testing was 224 × 224. During testing, both models applied multidimensional image re-sizing (multi cropped images) derived from 256 × 256, to improve the model’s performance by using images with different dimensions, allowing more visibility of the object features [6, 7].

### Results

#### Chemical crystal image database

A database consisting of 6994 images from the eleven chemical crystals was created and all images were used for training. For testing, the total number of crystal images was 180, 60 images for each repetition. To be used for testing, the unknown chemical images were saved outside the database, with each repetition in a respective folder.

All images were taken under the same resolution (3488 × 2616 pixels) and microscopic magnification (2.25× with a field of view of 8.9 mm). The only difference among them was light balance (brightness and contrast), caused by the nature of the crystal. Table 1 shows the distinctive visual characteristics of the images.

**Table 1:**
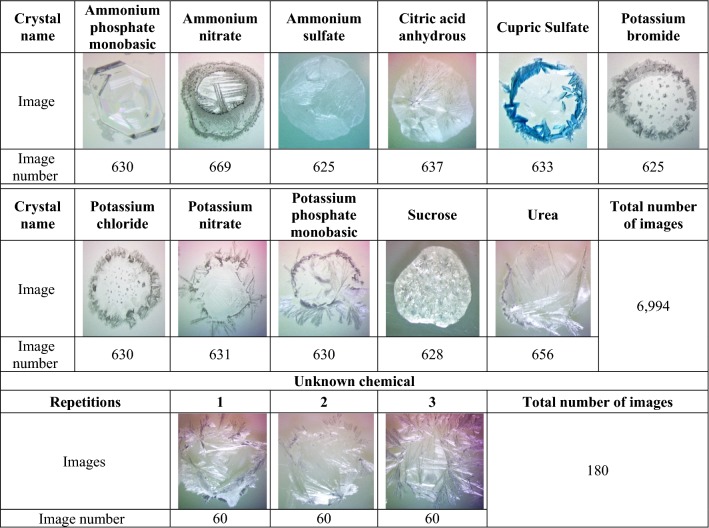
Database of crystal images and respective image number

#### Training results of GoogLeNet and VGG-16 models

**Fig. 1:**
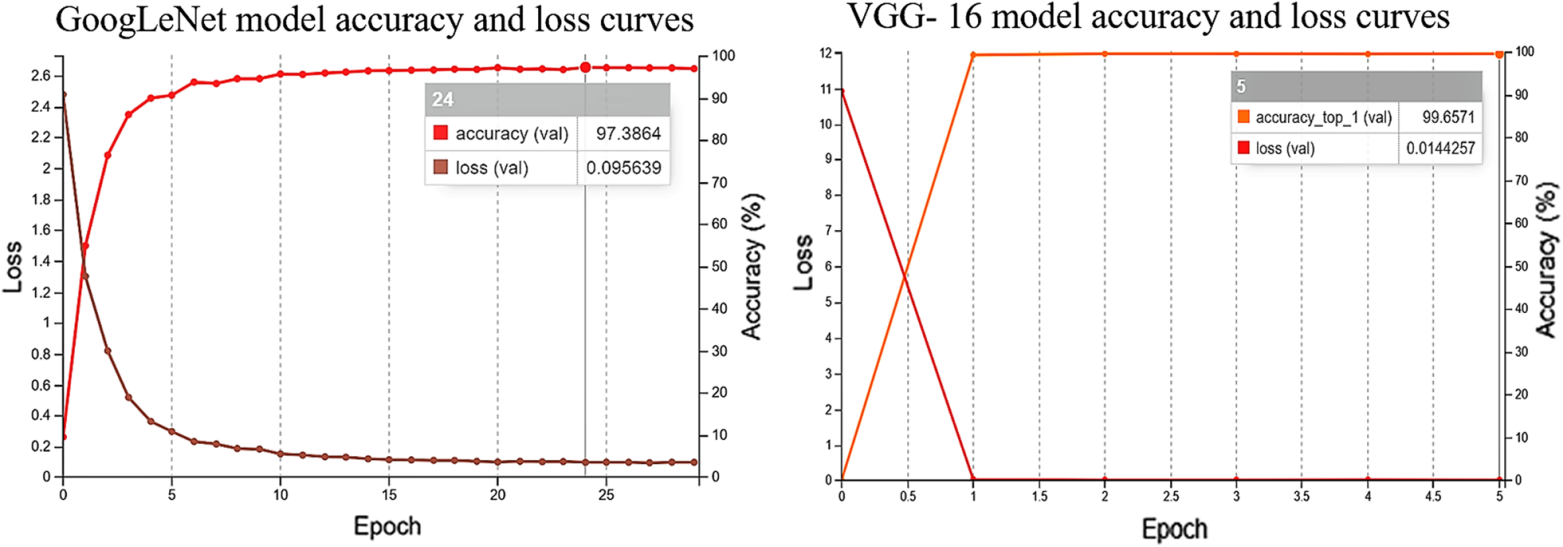
Training results for GoogLeNet and VGG-16 models, with accuracy (ascending curves) and loss (descending curves)

The results presented in Fig. 1, shows that both models achieved high accuracy values during training. The GoogLeNet model reached maximum accuracy value in training epoch 24, with validation accuracy percentage of 97.38% and a loss of 0.09. The VGG-16 model presented the most uniform accuracy and loss curves. From epoch 1, the model already presented accuracy values above 90%, after which it was aborted since no significant improvements in accuracy values were observed. The model reached the highest accuracy value of 99.65% and a minimum loss of 0.014. Epoch 24 for GoogLeNet and 5 for VGG-16 were chosen to test the model performance in image classification.

#### Probability and image classification results

**Fig. 2:**
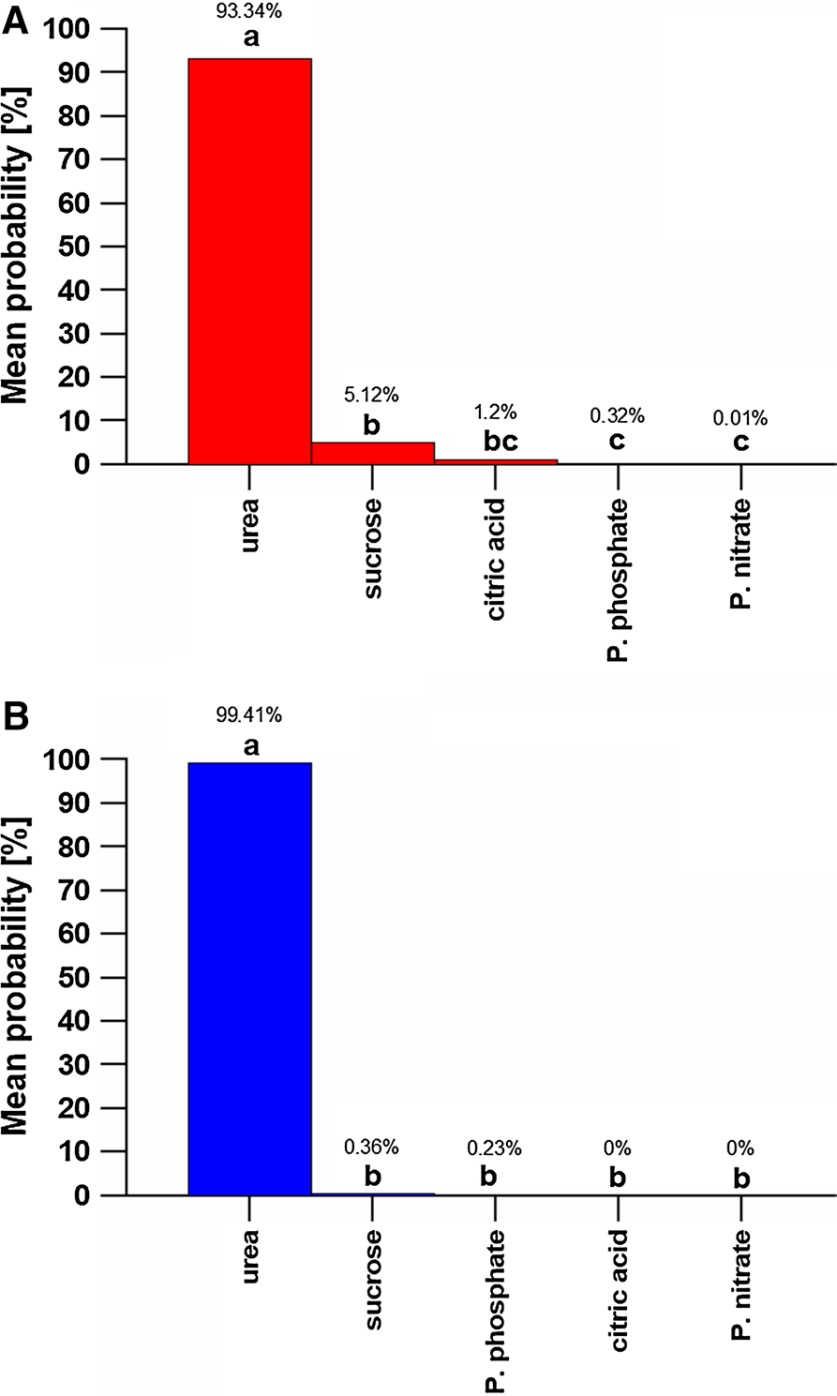
Probability analysis and image classification comparative results for **A** GoogLeNet and **B** VGG-16 models. Figure bars identified with different letters (a–c) are significantly different from each other

In the image classification and associated probability analysis both models presented similarities regarding the top five predicted classes, shown in Fig. 2. From the five classes predicted with highest probabilities by the GoogLeNet model, urea presented the highest average probability percentage of 93.34%, which was very highly significantly different (p < 0.001) from the remaining classes (Fig. 2A). Average probabilities for sucrose (5.12%), and citric acid (1.2%) were the second and third highest, respectively.

The VGG-16 model presented the same five highest-ranked chemical classes as GoogLeNet, but with a slightly different order (Fig. 2B). Urea, with an average probability of 99.41%, was again very highly significantly different (p < 0.001) from the remaining classes. Sucrose had the second-highest average probability ranking, but was very low (0.36%), and not significantly different from all the remaining lower-ranked classes (Fig. 2B). Both models identified the unknown chemical as urea with very high average confidence (> 93%, Fig. 2A, B). The similarities between urea used for training the models, and the unknown chemical crystals images are shown in Table 1.

### Discussion

The results obtained in this study shows excellent performance of both deep learning models in training and image classification. Thus, both models were able to identify urea as the unknown chemical. However, VGG-16 performed slightly better than GoogLeNet in both training and in image classification. In the classification phase (Fig. 2), VGG-16 achieved a higher average true positive result for urea (99.41%) than GoogLeNet (93.34%), and a lower false positive result for sucrose (0.36% and 5.12% for VGG-16 and GoogLeNet, respectively). According to Simonya and Zisserman [7], this improved performance of VGG-16 can be attributed to the initial layers with a small receptive field, which were designed to make the decision function more discriminative. Whereas, the GoogLeNet architecture starts with a big receptive field, intended to improve computer efficiency by decreasing the computation requirements [6]. From the ILSVRC14, Russakovsky et al. [5] reported that the GoogLeNet has more difficulties in recognizing small features or complex images, which agrees with our findings that GoogLeNet was less accurate in detecting microscopic crystalline structures.

Deep architectures are normally associated with improved accuracy and performance in image classification. Our study agrees with others including Simonya and Zisserman, and Fuentes et al., which found that VGG-16 outperformed deeper VGG models [7], as well as two other deep networks (ResNet-50 and ResNeXt-50) [4]. The GoogLeNet model, however, uses inception modules, which increases the efficiency of computer resources (time and energy, particularly with large datasets) with improved accuracy in image classification tasks.

## Conclusions

Both deep learning models yielded excellent results when using image classification to identify samples of different chemical crystals. Training accuracy values of 97.38% and 99.65% were obtained for GoogLeNet and VGG-16, respectively, showing that deep learning is a suitable method to identify materials and objects using digital light microscopy images. Both models used in this study recognized urea as the unknown chemical, with average probabilities ranging from 93.34 to 99.41%.

Based on the results of this study, deep learning machine vision has high potential to be used for the automated microscopic identification of other materials and small objects which can be imaged with light microscopy.

## Limitations

- Hazardous chemicals and those that react dangerously with water, or have high volatility, were not included in this study and are not recommended.
- Some hygroscopic chemicals were not included. These chemicals hardly get dry and absorb humidity in the environment too quickly.
- When the number of images and classes in the database is small, the models are unable to correctly classify the different categories.

## Abbreviations

CNN: convolutional neural networks
DIGITS: Deep Learning GPU Training System
GoogLeNet: Google Network
GPU: graphics processing unit
ILSVRC14: ImageNet Large-Scale Visual Recognition Challenge 2014
RGB: red, green and blue, color combination (true color)
ROI: region of interest
VGG-16: Visual Geometry Group with 16 weight layers
